# New Antibiotics for the Treatment of Acute Bacterial Skin and Soft Tissue Infections in Pediatrics

**DOI:** 10.3390/ph13110333

**Published:** 2020-10-23

**Authors:** Nicola Principi, Alberto Argentiero, Cosimo Neglia, Andrea Gramegna, Susanna Esposito

**Affiliations:** 1 Università degli Studi di Milano, 20122 Milan, Italy; nicola.principi@unimi.it; 2 Pediatric Clinic, Pietro Barilla Children’s Hospital, Department of Medicine and Surgery, University of Parma, 43121 Parma, Italy; alberto.argentiero@unipr.it (A.A.); cosimo.neglia@unipr.it (C.N.); 3 Fondazione IRCCS Ca’ Granda Ospedale Maggiore Policlinico, Internal Medicine Department, Respiratory Unit and Cystic Fibrosis Adult Center, 20122 Milan, Italy; andrea.gramegna@unimi.it; 4 Department of Pathophysiology and Transplantation, University of Milan, 20122 Milan, Italy

**Keywords:** acute bacterial skin and soft tissue infections, ceftaroline fosamil, children, methicillin-resistant *Staphylococcus aureus*, ozenoxacin

## Abstract

Acute bacterial skin and soft tissue infections (aSSTIs) are a large group of diseases that can involve exclusively the skin or also the underlying subcutaneous tissues, fascia, or muscles. Despite differences in the localization and severity, all these diseases are due mainly to Gram-positive bacteria, especially *Staphylococcus aureus* and *Streptococcus pyogenes*. aSSTI incidence increased considerably in the early years of this century due to the emergence and diffusion of community-acquired methicillin-resistant *S. aureus* (CA-MRSA). Despite the availability of antibiotics effective against CA-MRSA, problems of resistance to these drugs and risks of significant adverse events have emerged. In this paper, the present knowledge on the potential role new antibiotics for the treatment of pediatric aSSTIs is discussed. The most recent molecules that have been licensed for the treatment of aSSTIs include ozenoxacin (OZ), ceftaroline fosamil (CF), dalbavancin (DA), oritavancin (OR), tedizolid (TD), delafloxacin (DL), and omadacycline (OM). However, only OZ and CF have been licensed for use in children with aSSTIs, although the superiority of these antibiotics to those routinely used for the treatment of aSSTIs should be further demonstrated. Waiting for additional studies, OZ and CF should be prescribed for aSSTI treatment in the presence of the potential failure of old molecules.

## 1. Introduction

Acute bacterial skin and soft tissue infections (aSSTIs) are a large group of diseases that can involve exclusively the skin, such as in the case of impetigo, or have a deeper localization, with the involvement of the underlying subcutaneous tissues, fascia, or muscles, causing cellulitis, erysipelas, abscesses, and wound or burn infections [1]. The spectrum of aSSTI severity can significantly vary, as impetigo can spontaneously resolve or heal with topical treatment only, whereas deeper diseases are generally more severe and can become life-threatening when not adequately treated [2]. Despite differences in the localization and severity, all these diseases are united by the fact that they are due mainly to Gram-positive bacteria, especially *Staphylococcus aureus* and *Streptococcus pyogenes*, although in rare cases, particularly in patients with polymicrobial disease, Gram-negative rods can be detected [2].

The incidence of aSSTIs is high. In the USA, it has been calculated that several million people are admitted to the emergency department for an SSTI episode every year, and a non-marginal number of them are hospitalized [3]. Children are a relevant portion of people with aSSTI, as it has been reported that the annual number of individuals younger than 18 years who are seen in USA emergency departments for an aSSTI is slightly less than 400,000 [4], and that a significantly greater number of children visit the physician’s office and are not included in the official epidemiological evaluations [5]. The aSSTI incidence has not always been the same. It increased considerably in the early years of this century and then stabilized or even slightly decreased [6,7]. This increase has been related to the emergence and diffusion of community-acquired methicillin-resistant *S. aureus* (CA-MRSA) [8]. The stabilization or slight decrease in incidence has coincided with the availability of new protocols for aSSTI diagnosis and treatment and the availability of new antibiotics effective against CA-MRSA. It was recommended that, for the empiric coverage of CA-MRSA in the outpatient setting, topical mupirocin and retapamulin and oral clindamycin, trimethoprim-sulfamethoxazole, tetracycline (doxycycline or minocycline), and linezolid be prescribed. In hospitalized patients, it was suggested that, pending culture results, vancomycin, linezolid, daptomycin, telavancin, or clindamycin be used [9]. However, for all these drugs, some problems have emerged. The resistance of *S. aureus* to these drugs has been repeatedly reported. Moreover, the risks of significant adverse events have been evidenced. Among others, vancomycin can be nephrotoxic and necessitates drug-level monitoring [10,11], linezolid can be associated with the development of myelosuppression [12], and daptomycin can induce an increase in the creatine phosphokinase serum levels [13]. The drawbacks explain why new antibiotics against *S. aureus* have been developed [14,15]. Some of them have very interesting pharmacokinetic and microbiological characteristics. Unfortunately, as usually occurs for all developing drugs, most of the new antibiotics that have been licensed for use in adults have been poorly studied in children or have significant intrinsic limitations for their use in pediatrics. In this paper, the present knowledge on the potential role of these new antibiotics for the treatment of pediatric aSSTIs is discussed.

## 2. Topical Antibiotics

Topical treatment is indicated for impetigo, the most common aSSTI of children, particularly those 2–5 years old. Two types of impetigo exist: bullous and non-bullous. The first type, which is significantly less common, is always due to *S. aureus*. In contrast, in the etiology of the non-bullous type, together with *S. aureus*, which remains the most important pathogen (70% of cases), *S. pneumoniae* can also play a role, alone or in combination [16,17]. Topical treatment is recommended in patients with mild disease (lesions limited to no more than 100 cm^2^ in total area in adults and less than 2% of the total body surface in pediatric patients). In the first decades of the antibiotic era, antibiotics such as bacitracin, erythromycin, neomycin, and rifamycin were used. None of them are presently recommended for topical treatment due to the resistance of infecting pathogens and/or the risks of adverse events [18].

Preparations containing mupirocin (MU, 2%); retapamulin (RET, 1%); and, outside the USA, fusidic acid (FA, 2%) have been used for several years. MU is the major metabolite of *Pseudomonas fluorescens* fermentation and inhibits bacterial protein synthesis through the prevention of isoleucine incorporation into protein chains [19]. RET is a semisynthetic derivative of pleuromutilin, a naturally occurring tricyclic antibiotic that inhibits bacterial protein synthesis by binding to domain V of 23S rRNA and blocking peptide formation [20]. FA inhibits translocation during protein synthesis. It has been largely prescribed in Europe and in several other countries worldwide, but the recent evidence that up to 50% of *S. aureus* detected in impetigo cases were resistant to this drug has raised problems for its use [21]. Presently, MU 2% ointment is the most frequently prescribed preparation in many countries, as it can be administered to infants only 2 months old, is safe and well tolerated, is available as a brand and generic product, is relatively low cost and is generally effective after 5 days of administration [22,23,24,25,26]. 

Compared to MU, RET 1% cream has a similar efficacy but some limitations. It is licensed only for children aged 9 months or older, should not be prescribed in cases due to MRSA, and is significantly more expensive. Moreover, it is no longer licensed in Europe after the company that originally developed MU decided to cease the worldwide manufacture and supply of the drug [27]. However, in recent years, MU’s efficacy for impetigo treatment has been debated, in parallel with the increasingly widespread use of this antibiotic and the progressive emergence of CA-MRSA. The clinical relevance of this microbiological phenomenon cannot be precisely defined because, despite being detected worldwide, the prevalence of *S. aureus* resistance to MU significantly varies among geographic areas and among years of study, even in the same geographic area [28]. In the USA, a prevalence varying from 3.4% in 2009 [29] to 31.3% in 2013 [30] was reported. Greater variations were also evidenced in Europe, with a prevalence lower than 5% in France [31] and Belgium [32] and higher than 50% in the UK [33]. In general, resistance was more common among MRSA than among methicillin-susceptible *S. aureus* (MSSA), and although most of the data have been collected in adults, making the evaluation of the problem in children difficult; in some cases, resistance was high enough to strongly question the use of MU in the empirical treatment of pediatric impetigo. This issue explains why attempts to develop new antibiotics for the topical treatment of impetigo have been made (Table 1).

### Ozenoxacin (OZ)

Ozenoxacin (OZ) is the first example in this regard. It is a nonfluorinated quinolone that has been licensed for impetigo treatment in adults and children 2 months of age and older by the US Food and Drug Administration (FDA) in 2017 [34] and, more recently, by the European Medicines Agency (EMA) [35]. In vitro studies have shown that OZ is bactericidal against many Gram-positive bacteria, including MSSA, MRSA, *Staphylococcus epidermidis*, and *S. pyogenes*. Compared to FA, MU, and RET, OZ has been found to be significantly more effective, as the minimal inhibitory concentrations (MICs) for all the etiologic agents of impetigo, including MSSA and MRSA, were several times lower for OZ than for other antibiotics [36]. The risk of the emergence of resistance to OZ was considered lower than that of many other quinolones, as in vitro resistance studies have shown that the MICs for sensitive bacteria did not change when the drug was used against pathogens carrying mutations or certain efflux pump mechanisms usually associated with resistance to other quinolones [37]. OZ seems to have ideal molecular and pharmacokinetic characteristics for the topical treatment of impetigo. The OZ molecule does not contain fluoride and halogen substituents, which should lead to improved tolerability and increased safety compared to those of other quinolones [38]. Moreover, OZ is not absorbed. Patients of any age, including infants, exposed for a long time to topical OZ administration showed no or negligible plasma levels of the drug [39,40].

Experimental and human studies have confirmed the potential role of OZ in topical impetigo treatment. In a mouse model of skin dermal infection due to *S. aureus*, it was shown that the bacterial eradication rate after 1% OZ was significantly higher (53% and 47% with OZ cream and ointment, respectively) than that obtained with mupirocin or retapamulin (28% or 31%, respectively) [41]. However, the data collected in humans, despite clearly supporting that OZ was more effective than placebo for impetigo treatment, did not confirm the superiority of this drug over the other antimicrobial preparations. This result is evidenced by the analysis of the results of two phase 3 studies that had led to the licensing of OZ in the USA. The first trial [42] enrolled a group of patients with impetigo (79.3% with the non-bullous type), among whom 61% were children (at least 2 years of age). The aim was to evaluate the efficacy of OZ 1% cream (155 cases), RUT 1% ointment (154 cases), and placebo (156 cases) given 2 times per day for 5 days (a fingertip unit, approximately 0.5 g, over the affected skin). The total affected area had to be >1 cm^2^ but <100 cm^2^. The severity of the lesions was established using a seven-symptom (exudate/pus, crusting, erythema/inflammation, pain, itching, tissue warmth, and tissue edema) skin infection rating scale (SIRS), in which each symptom was scored from 0 (no symptom) to 6 (relevant severity). To be enrolled, a score of at least 8 had to be calculated. After enrolment, the efficacy was evaluated on days 3–4 during the 5-day study treatment, on days 6 or 7 (1–2 days after the end of study treatment), and on days 10–13 (5 to 8 days after the end of the study treatment) by measuring the variations in the SIRS score. Early cure and improvement were diagnosed when, after 3–4 days of therapy, the total SIRS score decreased >10%. At the end of therapy, the patients were considered cured when the SIRS score was 0 for exudates/pus, crusting, tissue warmth, and pain and no more than 1 each for erythema/inflammation, tissue edema, and itching. Subjects with a total SIRS score that decreased >10% and not fulfilling the criteria of individual SIRS scores for cure were considered only improved. Finally, the last visit was used to establish persistent cure or the appearance of relapse. When possible, at any visit, a microbiological evaluation of the etiology of skin lesions was performed. At enrolment, *S. aureus* was found alone or in association with other skin pathogens in 66.4% of the cases. However, the rate of MRSA was very low. The results showed that, although early evaluation did not show any difference in the OZ efficacy compared to that of the placebo, at the end of therapy the proportion of patients with a clinical cure was greater in the OZ group (35%) than in the placebo group (19%) (difference 15.5%; 95% confidence interval [CI], 5.6–25.5%; *p* = 0.003). Moreover, no additional antimicrobial therapy was necessary following the study treatment. The efficacy was greater in patients with non-bullous disease, a low SIRS score, and a smaller affected area. Moreover, OZ administration was associated with a better microbiological response. At the end of therapy, the eradication or presumed eradication of the infecting pathogen(s) was demonstrated in 79% of the OZ-treated patients compared to 57% of the controls, with a statistically significant difference between the groups (difference 27%; 95% CI, 18–37; *p* = ≤0.0001). Even if OZ was effective in cases due to *S. aureus*, the very low number of MRSA cases did not allow us to evaluate the effect of OZ on this pathogen. However, when the data regarding children receiving RET were analyzed, it was apparent that this drug was substantially as effective as OZ because 37.7% of the patients receiving retapamulin were cured at the end of treatment. A substantial equivalence between OZ and RET was also reported for the microbiological success rate. Although the prevalence of eradication was significantly higher at early evaluation in subjects treated with OZ, the difference was no longer evident at the end of therapy when a microbiological cure was demonstrated in 79.2% of the OZ cases and in 81.7% of the patients in the RET group. The safety and tolerability of the OZ preparation was very good as, in the study, only approximately 3% of patients had treatment-emergent adverse events, a value quite similar to that reported in the retapamulin and placebo groups. All the adverse events were mild and spontaneously resolved in a few days, confirming what had already been reported in some phase 1 OZ trials [43,44].

The second trial aimed to compare OZ with a placebo. No internal validity arm was included. However, the methods used to conduct the study were quite similar to those used for the first study. The only exception was that the minimum age was two months, the minimum total affected area was 2 cm^2^, and a five-symptom SIRS was used to determine the lesion severity (blistering, exudate and/or pus, crusting, erythema and/or inflammation, and itching and/or pain on a scale of 0 (absent) to 3 (severe), with a minimum total score of 3 [45]). A total of 206 patients were treated with OZ, and the same number of patients was given placebo. More than 60% in both groups were children. *S. aureus* was the predominant pathogen (54%). Therapy was considered effective when the score was 0 for blistering, exudate and/or pus, crusting, and itching and/or pain and ≤1 for erythema and/or inflammation. After 2 days of therapy, clinical success, defined as early cure/improvement, was achieved in a greater number of subjects given OZ than among the controls (75.7% vs. 59.2%). The clinical superiority of OZ over a placebo was confirmed at the end of 5 days of therapy. when cure was evidenced in 54.4% of cases vs. 37.9% (*p* = 0.001) and some days after treatment discontinuation when cure/improvement were ascertained in 88.8% and 78.2%, respectively (*p* = 0.003). The analysis of skin cultures revealed that the OZ administration was more effective than the placebo in the elimination of infecting bacteria. Two days after the start of therapy, the cultures were negative in 87.2% of treated patients compared to 63.9% of controls (*p* = 0.002). Similar differences were shown at the end of therapy. when the culture was negative in 92.0% of the OZ-treated patients and in 73.1% of the controls (*p* = 0.005). Combined clinical and microbiological responses were obtained in 57.6% of the OZ-treated group and in 34.5% of the placebo-treated subjects (*p* = 0.001).

Starting from this evidence, it can be concluded that OZ is effective in the treatment of impetigo, although no definitive conclusion can be drawn on the importance of OZ for the treatment of cases due to *S. aureus* strains resistant to currently used antibiotics, including MU. Even if, in the second of the above-described studies, 10 patients in the OZ group were infected with MU-resistant *S. aureus* strains and were fully cured by antibiotic administration, the total number of impetigo cases due to difficulty eradicating *S. aureus* is too low to affirm that OZ must be used instead of the presently used antibiotics in the topical treatment of impetigo. On the other hand, both the real incidence of MRSA as a cause of impetigo and the clinical implications of the resistance of skin pathogens to MU have not been precisely defined [46]. On the other hand, prolonged OZ administration has been associated with an increased risk of fungal or bacterial superinfection [47] that further supports the need for reliable studies before the official recommendations for the topical treatment of impetigo are modified. Awaiting further information, also considering the OZ costs in comparison to those of MU and other topical antibiotics [48], OZ must be limited to cases refractory to traditional topical treatment or caused by multidrug-resistant pathogens.

## 3. Oral and Parenteral Antibiotics

Table 2 summarizes new oral and parenteral antibiotics for the treatment of aSSTIs in pediatrics.

### 3.1. Ceftaroline Fosamil

Ceftaroline fosamil (CF) is a fifth-generation cephalosporin with interesting microbiological characteristics. It has high affinity for mutated penicillin-binding proteins that make bacterial pathogens resistant to other β-lactam antibiotics, thus becoming effective against MRSA. Moreover, it is active against vancomycin-intermediate *S. aureus* and some Gram-negative rods, excluding those producing extended-spectrum β-lactamases or carbapenemases [49,50]. Finally, the in vitro studies seem to indicate that bacteria exposed to CF have a low propensity to develop resistance [51,52], although the emergence of resistant strains during treatment has been described [53,54]. This antibiotic is licensed by the FDA for the treatment of community acquired pneumonia and aSSTIs in adults and children 2 months of age and older [55] and by the EMA [56] for adults and children of any age, including neonates.

Studies carried out in adults with aSSTI, in some cases due to MRSA, have shown that CF is not inferior to vancomycin plus aztreonam but assures a more rapid response [57]. At day 3 of therapy, the cessation of infection spread and the absence of fever were achieved in 74.0% of cases in the CF group and in 66.2% of those treated with control antibiotics (difference, 7.8%; 95% CI, 1.3–14.0%) [58]. Moreover, CF was found to be safe and well tolerated, with only 4% of patients showing mild and spontaneously resolving adverse events [59]. 

Experience with CF in children is scarce but enough to evaluate its safety and efficacy and establish the most appropriate dosage for any pediatric age. Combining the data of 5 studies in which information on CF pharmacokinetics was obtained [60,61,62,63,64], which pediatric doses produced an exposure to the antibiotic similar to that obtained in adults with the administration of 600 mg two times per day was established and found to be clinically effective. In particular, it was evidenced that, with a dosage regimen of 8 mg/kg every 8 h in children aged 2 months to <2 years and 12 mg/kg (up to a maximum of 400 mg) every 8 h in children aged 2 years to <18 years or 600 mg two times per day in children aged 12 to <18 years, >90% of children could have serum drug concentrations higher than the MIC of MRSA for more than 35% of the time between doses. Eleven preterm neonates with a gestational age varying between 32 and 37 weeks were included in the analysis, and the results of simulations suggested that a dosage of 6 mg/kg every 8 h would be adequate in infants <2 months of age. As CF is a time-dependent antibiotic, this result was considered highly indicative of the potential efficacy of the drug against MRSA-caused aSSTIs [65]. These dosages were found to be effective and safe in children with aSSTIs by Korczowsky et al. [62]. These authors compared CF (107 patients) with vancomycin or cefazoline plus aztreonam (52 patients) in children 2 months to 17 years old. The clinical cure rates were higher among CF-treated patients (94%) than among those receiving control therapy (87%). For patients with MRSA, the eradication of the infecting pathogen was achieved in 89% of the CF-treated cases compared to 57% of the control-treated cases. Interestingly, CF was effective in nine patients who had previously received an ineffective treatment, suggesting the potential use of this cephalosporin in difficult-to-treat cases. The rates of study drug-related treatment-emergent adverse events were similar for CF (22%) and a comparator (23%), although one serious adverse event (hypersensitivity) was diagnosed in a child receiving CF. 

However, the superiority of CF over other anti-*S. aureus* antibiotics cannot be considered fully demonstrated, as the data collected in comparative studies are too scarce to draw definitive conclusions. Even in this case, further studies, particularly in younger children, are needed.

### 3.2. Lipoglycopeptide Antibiotics

Lipoglycopeptide antibiotics are derivatives of the glycopeptides vancomycin and teicoplanin with improved antimicrobial activity and, at least in some cases, with different pharmacokinetic characteristics. The modifications of the heptapeptide core of glycopeptides with the addition of a lipophilic side chain and other minor molecular variations are the basis for these differences [66]. The most advanced lipoglycopeptide antibiotics with improved characteristics are dalbavancin (DA) and oritavancin (OR), which are approved by the FDA and EMA for the treatment of adults with complicated aSSTIs [67]. Both of these drugs are given intravenously and are 4- to 8-fold more potent than vancomycin against Gram-positive bacteria, including vancomycin-intermediate and vancomycin-resistant *S. aureus*. Moreover, they have a very high protein-binding affinity (>90%) and a very long half-life (approximately 2 weeks in healthy adults), which have led to the recommendation of a two-dose regimen initially and a one-dose regimen later [67,68] and support their use as outpatient treatment. 

Several studies have measured the clinical efficacy of DA and OR in patients with complicated aSSTIs by comparing these drugs with standard care (i.e., vancomycin or its traditional alternatives, such as linezolid, tedizolid, daptomycin, clindamycin, trimethoprim-sulfamethoxazole, doxycycline, oxacillin, cefazolin, CF, and tigecycline) [68,69,70,71,72]. A recent meta-analysis of randomized controlled trials, including those with two and only one dose of antibiotics [73], reported that the clinical response to lipoglycopeptide administration was similar to that to standard care, without differences between DA and OR. Clinical cure and pathogen eradication occurred in more than 90% of cases, even when MRSA was the causative pathogen. The incidence of serious adverse events following antibiotic administration was low and similar in patients receiving newer lipoglycopeptides and in those given traditional therapies. However, the overall adverse event incidence was lower in patients receiving dalbavancin than in controls (0.77; 95% CI, 0.64–0.93). 

The cost of therapy was lower with the use of each of the new drugs compared to with the use of vancomycin-based regimens. It was calculated that, for each treated patient, the administration of DA could save $1442 to $4803, while the use of OR could save $3571 to $6932. Economic advantages were ascribed mainly to the reduction in treatment duration. Compared to the standard of care, DA and OR could save between 6.5–10.0 and 7.5–11.0 days of treatment, respectively. This benefit is because their pharmacokinetic characteristics allow a one-time dosing regimen, contrary to what is needed when traditional antibiotic alternatives, including vancomycin, are used. The extreme simplification of the therapy makes these drugs very attractive for the treatment of aSSTIs in the ambulatory setting and emergency room, provided that the patients can be carefully followed up at home [74]. The administration of a single antibiotic dose eliminates prolonged parenteral therapy; reduces resource utilization; improves compliance; and, because it does not need a peripherally inserted central catheter, lowers the risk of vascular complications and infections. 

However, presently, neither DA nor OR can be used in children due to a lack of randomized clinical trials showing the efficacy and safety of these drugs in the pediatric population. For DA, some studies regarding pharmacokinetics and safety in children 3 months and older are available. They can be considered the prerequisite for clinical trials. It was shown that, to achieve a drug exposure similar to that obtained in healthy adults with a single dose of 1500 mg, doses of 18 mg/kg and 22.5 mg/kg were needed in subjects aged 6 to <18 years and 3 months to <6 years, respectively [75,76]. Adverse events occurred rarely, were mild, and disappeared spontaneously in a few days. A phase 1 trial (NCT02688790) with the aim of evaluating the pharmacokinetics and safety of a single dose of 22.5 mg/kg DA in neonates and infants <3 months has been recently terminated, but no results have yet been posted. The clinical evaluation of DA efficacy in children with a phase 3 trial is presently ongoing (NCT02814916).

OR has not been extensively studied in children. Only a phase 1 study with the purpose of evaluating the pharmacokinetics, safety. and tolerability of this drug in patients <18 years old with a confirmed or suspected bacterial infection is presently ongoing (NCT02134301). No results have been reported to date.

Both these new lipoglycopeptide antibiotics require further studies before they can be considered to treat aSSTIs in children.

### 3.3. Tedizolid (TD)

Tedizolid (TD) is an oxazolidinone antibiotic that is significantly more effective than linezolid against several Gram-positive strains, including MSSA, MRSA, and vancomycin-resistant *S. aureus*. Moreover, it is effective against the same *S. aureus* strains that are resistant to linezolid [77,78]. Furthermore, compared with linezolid, TD has more favorable pharmacokinetic characteristics. Its half-life is approximately double, which allows for once-daily dosing. Finally, it seems safer as, although TD is a more potent inhibitor of mitochondrial protein synthesis than linezolid, the risks of adverse effects related to mitochondrial dysfunction (i.e., lactic acidosis, myelosuppression, and neuropathy) were found to be inferior to those for linezolid in studies carried out in animal models [79,80]. TD is approved for the treatment of aSSTIs by the FDA [81] and EMA [82]. However, compared to linezolid, TD is significantly less expensive. It has been calculated that treatment with TD would cost about 50% less than that with linezolid [83]. Oral and intravenous formulations are available.

Experience of their use in children is very poor. A study to evaluate the pharmacokinetics of TD in children under 2 years old is ongoing (NCT03217565). Two clinical trials compared the efficacy of TD and linezolid for the treatment of aSSTI, mainly cellulitis/erysipelas, in adults [84]. A total of 644 patients received tedizolid (200 mg once daily for 6 days), whereas 669 were given linezolid (600 mg twice daily for 10 days). In the first study, antibiotics were given orally. In the second study, the doses of the first day of therapy were given intravenously, and from the second day therapy was administered by mouth. *S. aureus* was the most common infecting pathogen in both groups (>80%). Approximately 35% of isolates were MRSA. The clinical and microbiological efficacy were evaluated at the end of therapy and 7–14 days later. Both treatments were very effective, as more than 85% of patients in both groups had favorable outcomes, with the eradication of the pathogen regardless of the type of aSSTI and infectious agent. 

However, once again available data do not demonstrate the superiority of the new antimicrobial agent over older antistaphylococcal antibiotics. Further studies are needed to solve this problem. The potential utilization in children must be completely studied.

### 3.4. Delafloxacin (DL)

Delafloxacin (DL) is an anionic quinolone that is significantly more active than other quinolones against several Gram-positive and Gram-negative bacteria. In particular, against MRSA, DL is 32 times more active than levofloxacin [85]. This drug, available in oral and IV preparations, has been approved by the FDA [86] for the treatment of both pneumonia and aSSTIs and by the EMA for aSSTIs [87] in adults.

The clinical efficacy of DL in aSSTIs has been evaluated in two multicenter studies in which DL has been compared to vancomycin plus aztreonam [85]. Moreover, the microbiological efficacy of DL was compared to that of levofloxacin and ciprofloxacin. Although more than 30% of detected *S. aureus* was resistant to the other quinolones, DL had a very low MIC (0.25 μg/mL) against levofloxacin-nonsusceptible *S. aureus*, MSSA, and MRSA. Treatment with DL resulted in the eradication of S. aureus in 98.4% of treated patients, regardless of the susceptibility of *S. aureus*. 

However, no study has been carried out in children. This lack of research is because quinolones are contraindicated in pediatric patients owing to the supposed risk of developing severe musculoskeletal disorders, as demonstrated in juvenile animals [88].

### 3.5. Omadacycline (OM)

Omadacycline (OM) is a novel tetracycline that, through modifications on the tetracycline D-ring, has become able to overcome the common tetracycline resistance mechanisms, including efflux and ribosomal protection [89]. Together with some Gram-negative rods and atypical bacteria, OM is active against several multidrug-resistant Gram-positive organisms, including MRSA [90], and has been approved by the FDA [91] but not the EMA [92] to treat community-acquired bacterial pneumonia and aSSTIs in adults. Both oral and IV formulations are available. Studies in patients with aSSTIs (wound infection 46.8%, cellulitis/erysipelas 30.5%, and major abscess 22.7%) due to MRSA in 32.4% of the cases showed that OM was as effective as linezolid in both early clinical response and post-treatment evaluation [93]. Early clinical response, defined as patient survival, with a reduction in lesion area of ≥20% vs. baseline and no receipt of rescue antibacterial therapy was evidenced in 86.2% and 83.9% of patients, respectively (difference 2.3; 95% CI, −1.5 to 6.2). A successful clinical response at 7–14 days after the last dose of both drugs, defined as the patient being alive, with the resolution of the signs and symptoms of infection such that further antibacterial treatment was not needed, was achieved in 85.1% vs. 82.1% of the cases (difference 2.9; 95% CI, −1.0 to 6.9), regardless of the type of infection and the infecting organism. The incidence of side effects was similar and was 51.1% in patients receiving OM and 41.2% in those receiving linezolid. The side effects were described as mild to moderate and, as far as OM is concerned, were nausea and vomiting. However, only 0.3% of the treated patients discontinued the therapy due to side effects. Moreover, none of them was the cause of death.

No studies regarding children have been planned. On the other hand, considering that tetracyclines are not licensed for use in children <8 years due to the risk of adverse events, it is highly likely that this drug will not be developed for use in pediatrics.

## 4. Conclusions

The sensitivity of *S. aureus* to antibiotics is limited in time. After a period of use, all antibiotics that initially appear effective on all or very large proportions of *S. aureus* strains become progressively less able to inhibit *S. aureus* growth until they can no longer be considered possible therapeutic options for the treatment of those diseases. This phenomenon explains why new antibiotics effective against *S. aureus* are continuously developed and clinically evaluated. The most recent molecules that have been licensed for the treatment of aSSTIs include OZ, CF, DA, OR, TD, DL, and OM. Unfortunately, children are excluded from the use of the majority of these antibiotics. Some of them, such as DL and OM, belong to antibiotic classes for which use in pediatrics is contraindicated by the possible development of severe adverse events. Other antibiotics, such as DA, OR, TD, and DL, despite interesting microbiological and/or pharmacokinetic characteristics, have not been adequately studied in children. The real efficacy, safety. and tolerability of these drugs in children are totally unknown, and only further studies can reveal the potential benefits in pediatrics. Only OZ and CF have been licensed for use in children with aSSTIs. However, it must be highlighted that the superiority of these antibiotics to those used for the treatment of aSSTIs should be further demonstrated. While waiting for additional studies able to define the true impact of OZ and CF in pediatrics, these antibiotics should be prescribed for aSSTI treatment in the presence of the potential failure of old molecules.

## Figures and Tables

**Table 1:** Topical antibiotics for the treatment of impetigo.

Antibiotic	Advantages	Limitations
Mupirocin 2%	Safe and well tolerated, low cost, generally effective after 5 days of administration.	Not always effective against MRSA.
Retapamulin 1%	Similar efficacy to mupirocin 2%.	Licensed only for children ≥9 months, not recommended for MRSA, expensive, no longer licensed in Europe.
Fusidic acid 2%	Safe and well tolerated, low cost.	*S. aureus* resistance to this drug in 50% of impetigo cases, not available in USA.
Ozenoxacin	More effective against MSSA and MRSA in comparison with other topical antibiotics.	Increased risk of fungal or bacterial superinfection, high cost.

MRSA, methicillin-resistant *Staphylococcus aureus*; MSSA, methicillin-susceptible *Staphylococcus aureus.*

**Table 2:** New oral and parenteral antibiotics for the treatment of acute bacterial skin and soft tissue infections in pediatrics.

Antibiotic	Advantages	Limitations
Ceftaroline fosamil	Effective against MRSA. vancomycin-intermediate *S. aureus* and some Gram-negative rods, excluding those producing extended-spectrum β-lactamases or carbapenemases.	Few data on its superiority over other anti-*S. aureus* antibiotics in pediatrics.
Lipoglycopeptide (dalbavancin and oritavancin)	4- to 8-fold more potent than vancomycin against Gram-positive bacteria, including vancomycin-intermediate and vancomycin-resistant *S. aureus*; very high protein-binding affinity (>90%); very long half-life.	Lack of randomized clinical trials showing the efficacy and safety of these drugs in the pediatric population.
Tedizolid	Significantly more effective than linezolid against several Gram-positive strains, including MSSA, MRSA, and vancomycin-resistant *S. aureus*; more safe and less expensive than linezolid.	Little experience in children.
Delafloxacin	Significantly more active than other quinolones against several gram-positive and gram-negative bacteria	No study has been carried out in children.
Omadacycline	Novel tetracycline active against some Gram-negative rods; atypical bacteria; and several multidrug-resistant Gram-positive organisms, including MRSA.	No studies regarding children have been planned. Considering that tetracyclines are not licensed for use in children <8 years due to the risk of adverse events, it is highly likely that this drug will not be developed for use in pediatrics.

MRSA, methicillin-resistant *Staphylococcus aureus*; MSSA, methicillin-susceptible *Staphylococcus aureus.*
